# Mastacembelid eels support Lake Tanganyika as an evolutionary hotspot of diversification

**DOI:** 10.1186/1471-2148-10-188

**Published:** 2010-06-19

**Authors:** Katherine J Brown, Lukas Rüber, Roger Bills, Julia J Day

**Affiliations:** 1 Department of Genetics, Evolution & Environment, University College London, Wolfson House, 4, Stephenson Way, London NW1 2HE, UK; 2 Department of Zoology, The Natural History Museum, Cromwell Road, London SW7 5BD, UK; 3 South African Institute for Aquatic Biodiversity, Grahamstown, South Africa

## Abstract

**Background:**

Lake Tanganyika (LT) is the oldest of the African Rift Lakes and is one of the richest freshwater ecosystems on Earth, with high levels of faunal diversity and endemism. The endemic species flocks that occur in this lake, such as cichlid fishes, gastropods, catfish and crabs, provide unique comparative systems for the study of patterns and processes of speciation. Mastacembelid eels (Teleostei: Mastacembelidae) are a predominately riverine family of freshwater fish, occurring across Africa and Asia, but which also form a small species flock in LT.

**Methods:**

Including 25 species across Africa, plus Asian representatives as outgroups, we present the first molecular phylogenetic analysis for the group, focusing particularly on the evolutionary history and biodiversity of LT mastacembelid eels. A combined matrix of nuclear and mitochondrial genes based on 3118 bp are analysed implementing different phylogenetic methods, including Bayesian inference and maximum likelihood.

**Results:**

LT *Mastacembelus *are recovered as monophyletic, and analyses reveal the rapid diversification of five main LT lineages. Relaxed molecular clock dates provide age estimates for the LT flock at ~7-8 Myr, indicating intralacustrine diversification, with further speciation events coinciding with periods of lower lake level. Our analyses also reveal as yet undescribed diversity of lacustrine and riverine species. A Southern-Eastern African clade, that is younger than the LT flock, is also recovered, while West African taxa are basal members of the African mastacembelid clade.

**Conclusions:**

That the LT species flock of mastacembelid eels appears to have colonised and immediately diversified soon after the formation of the lake, supports the view of LT as an evolutionary hotspot of diversification. We find evidence for biogeographic clades mirroring a similar pattern to other ichthyological faunas. In addition, our analyses also highlight a split of African and Asian mastacembelid eels at ~19 Myr that is considerably younger than the split between their associated continents, suggesting a dispersal scenario for their current distribution.

## Background

The African Great lakes provide natural experimental settings in which to better understand the processes that underlie speciation. Lake Tanganyika (LT), the oldest African rift lake (9-12 Myr) [1], is one of the world's richest freshwater ecosystems (c. 2000 species) [2]. It harbours numerous different endemic faunas (c. 600 species) [2], supporting more endemic non-cichlid species than any of the other African Great Lakes, many of which form evolutionary radiations, termed 'species flocks' [3]. The most impressive of these are represented by cichlid fishes [4]; however, unique to LT, and possibly a consequence of its older geological history, are the multiple radiations, that have evolved from a variety of taxonomic lineages, e.g., gastropods, crabs, catfish, spiny-eels, sponges, atyid prawns and ostracods. Molecular phylogenetics and molecular dating techniques have enabled inferences to be made of colonisation and diversification histories, which have alternatively supported the perception of LT as a hotspot of diversification [5-7], as well as an evolutionary reservoir [8,9]. The existence of multiple radiations of unrelated faunas within an island-like setting that display differing life histories and ecologies, offers a unique comparative opportunity to study the dynamics of radiations and the importance of this lake as a cradle of speciation. Through this system we can begin to ascertain the relative importance of extrinsic versus intrinsic processes in the context of adaptive radiation theory [10,11].

Species diversification within LT has manifested in the form of large scale super-flocks such as the well-studied cichlid fishes containing ~200-250 species [12,13] as well as the thiarid gastropods [14], containing ~70 species. However, the majority of taxa form small-scale faunal radiations at the generic level, containing between 10-15 species. Because of the high levels of interest in cichlid fishes as model organisms in which to investigate speciation processes, non-cichlid flocks have been largely overlooked. Recently, however, different authors have begun to address this from a molecular phylogenetic perspective to determine timing of diversification, as well as colonisation history, e.g. *Synodontis *catfish [7,15,16], *Platythelphusa *crabs [6], and thiarid gastropods [9,14].

To-date, the evolutionary history of the LT species flocks reveals both similarities and differences, although inconsistencies as a consequence of molecular dating may exacerbate these disparities. For example, LT cichlid tribes (based on fossil calibrations [12]), *Synodontis *catfish [7,15], *Platythelphusa *crabs [6] and *Lavigeria *gastropods [9] all show within-lake diversification supporting the notion of LT as a hotspot of diversification. In contrast, the majority of gastropod diversity found within LT has evolved from lineages that predate its formation [9]. A similar pattern was also hypothesized for the LT cichlid fish tribes, indicative of multiple independent colonisation events into LT [8,17]. This scenario is further supported if Gondwanan vicariance dates are enforced to calibrate molecular time estimates [12,18]. However, that colonisation history differs between faunal groups appears largely to be a consequence of comparing different taxonomic units. *Platythelphusa *crabs represent a single colonisation event followed by subsequent diversification [6], while *Synodontis *also form a flock within LT, but with the inclusion of a non-endemic species that appears to represent evolution within the lake followed by emigration [15]. That these unrelated groups exhibit different evolutionary histories makes the analysis of other taxonomic groups, using robust phylogenetic methods important in furthering the understanding of the role of LT as a potential diversification hotspot.

Mastacembelids or spiny-eels (Teleostei: Synbranchiformes) is a predominately riverine family [19], with an Old World distribution throughout tropical Africa and Asia (~78 species), although the majority of species occur in Africa (68%). Based on morphology, there is little evidence for the separation of mastacembelids into two subfamilies [20], or African species into three genera [21-23] and therefore we refer to all African species as *Mastacembelus*. Little is known about mastacembelids in terms of their phylogeny, ecology and life history, and this is even more apparent in the species that have formed a radiation within LT. This is, in part, because of their cryptic and predominately rock-dwelling nature making them difficult to study. Aside from the LT radiation, the only other African region with a comparable number of endemic sympatric species is in the lower Congo River [24], making these species assemblages of interest with regards to the factors promoting and maintaining elevated levels of endemicity. Here we focus on the LT species flock.

There are currently 13 described mastacembelid species endemic to LT [25], as opposed to a single (possibly two) endemic species within Lake Malawi [26,27]. This asymmetry is also seen in other groups that form radiations in Tanganyika but not Malawi (e.g. *Synodontis *catfish), although notably Lake Malawi *Bathyclarias *catfish form a small species flock [28]. Despite the age and size of Lake Malawi, and the fact that, like LT, it supports a large-scale radiation of cichlids [29], this asymmetry between the two lakes in species diversity of *Mastacembelus *and *Synodontis *is noteworthy. Potential factors, such as the repeated periods of desiccation experienced in Lake Malawi [30,31], or niche availability with the presence of an extensive cichlid radiation, may have impinged on the abilities of other faunas to diversify.

Here, using a multigene dataset of mitochondrial (mtDNA; Cytochrome *b *[Cyt *b*], Cytochrome *c *oxidase sub-unit 1 [CO1]) and nuclear (ncDNA; ribosomal S7 introns 1 and 2 [S7]), and several methods of phylogenetic inference and relaxed-clock dating we present the first molecular phylogenetic analysis of *Mastacembelus *eels (Additional file 1, Table S1). We focus on LT species, to investigate their diversity, monophyly and colonisation history, in order to infer whether small-scale radiations are more likely to diversify in intralacustrine conditions, as opposed to having diversified outside of the lake basin, and discuss our results in a comparative framework. As such the majority of sampling is from LT (including 11 of the 13 currently described species) and associated catchments. In addition almost half of all described African mastacembelid diversity is included, along with two Asian mastacembelid species as outgroups, in order that we can evaluate the LT species flock in the broader context of mastacembelid biogeography and evolutionary history.

## Results and Discussion

### Sequence analysis

The preferred evolutionary models, calculated using Modeltest v3.7 [32], are GTR+I+Γ for the Cyt *b *datatset, HKY+I+Γ for both S7 and CO1 datasets, and TrN+I+Γ for the concatenated dataset. The nucleotide base composition (A: C: G: T) for each molecular marker is as follows: 27.5: 36.1: 11.6: 24.8% (Cyt *b*), 26.9: 18.4: 24.4: 30.3% (S7), and 31.0: 30.3: 12.8: 25.9% (CO1). The bias against guanine in Cyt *b *has also been reported in other fish taxa [33]. The χ^2 ^test of homogeneity demonstrated that there was no significant difference in base frequencies between ingroup taxa (χ^2 ^values of 103.5, 39.1 and 40.8, with 231, 210 and 240 degrees of freedom for Cyt *b*, S7 and CO1 respectively, p < 0.05). The data were combined in a total evidence approach, with the total matrix consisting of 3118 bp [1206 bp from Cyt *b *(of which 70 bp from tRNA); 1129-1162 bp (1224 bp aligned) from S7 (1st intron 853-906 bp, 2nd intron 233-263 bp); and 688 bp from CO1]. Data were missing for the 2nd intron of S7 for 12 specimens.

### Phylogenetic relationships and biodiversity

The Maximum-likelihood (ML) and Bayesian (BI) trees generated from the concatenated dataset resolved virtually identical topologies, with neither tree being assigned as a better fit to the data than the other (AU test, p = 0.500). The concatenated and single gene datasets also yielded largely congruent tree topologies (see Additional files 2, 3 and 4 for individual gene trees), although all individual datasets demonstrated a significantly worse fit to the data when compared to the concatenated dataset (AU test, p = 0.008 for Cyt *b*, and p = 0.000 for both S7 and CO1 BI trees). CO1 performed well for species-level identifications, but sister species and deeper-level relationships were poorly supported, making it of limited use for phylogenetic analysis. In terms of the use of CO1 for DNA taxonomy, it faired less well in terms of resolution and support than Cyt *b *for *Mastacembelus*. This lack of resolution between deeper nodes was also reported with CO1 in LT thiarid gastropods, with this being attributable to either the marker evolving too rapidly for resolving deeper systematic levels, or the rapid simultaneous evolution of major clades resulting in similar levels of sequence divergence [14].

**Figure 1:**
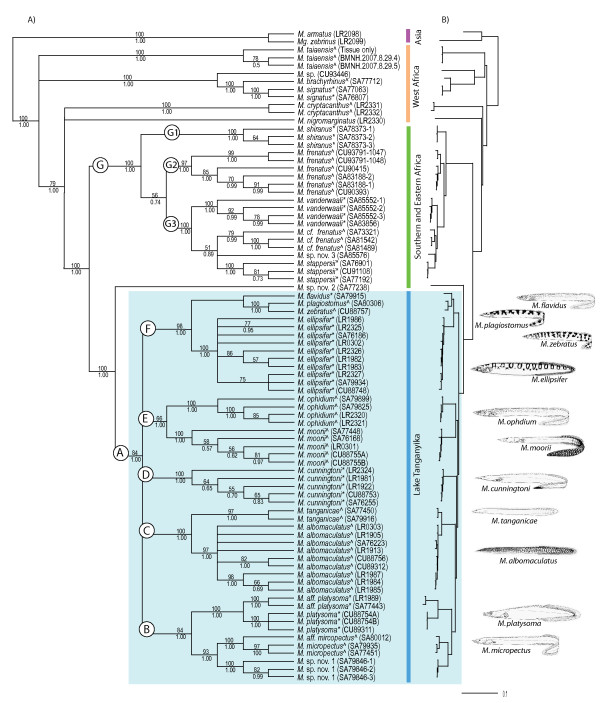
**Phylogenetic relationships of African mastacembelid eels inferred from the concatenated mtDNA and ncDNA datasets**. A) The consensus of the ML and BI trees, with support from bootstrap generated from ML and Bayesian posterior probability (BPP), above and below branches respectively (shown for nodes with greater than 50% support only). Key nodes are labelled A-G, with the blue box highlighting LT species. B) Phylogram based on Bayesian analysis of concatenated data set (mtDNA and ncDNA) to depict branch lengths. *M., Mastacembelus; Mg., Macrognathus*. Generic classification following Travers [21,23] represent *Aethiomastacembelus *(*) and *Caecomastacembelus *(^). Pictures are reproduced from Eccles [96].

Support for sister species relationships was also lower in the ncDNA S7 than Cyt *b *single gene analyses. It is possible that S7 could be evolving too slowly to resolve the sister species relationships within LT. This is also the case for *Synodontis *[15], despite this gene having proved useful in elucidating cichlid relationships of a similar age [34]. Combining the data into one concatenated dataset improved support in all parts of the tree (Fig. 1).

Our analyses recover a well-supported LT *Mastacembelus *flock (node A, Fig. 1), composed of five main lineages (nodes labelled B-F, Fig. 1). However, while relationships within these lineages are well supported, the relationships between them are poorly supported, resulting in a basal polytomy. The use of more rapidly evolving markers (e.g. AFLPs) is likely to further elucidate the relationships between species within the LT radiation, as demonstrated for rapidly speciating clades [35-38]. If, however, the data do represent a true hard polytomy, we interpret this result to suggest early rapid speciation of this clade in to new available niches, indicative of an adaptive radiation [10]. Short branches are also found within the *Platythelphusa *crabs [6] and LT *Synodontis *[7,15] also indicating a similar rapid diversification in these clades.

Molecular phylogenetic analyses reveal greater diversity of the spiny-eel species flock than morphological studies, with 13 species recovered. Our analyses reveal cryptic diversity within *M. platysoma*, forming two distinct clades, pertaining to specimens occurring in the northern (*M. platysoma*; Kigoma, Tanzania) and southern (*M. aff. platysoma*; Mpulungu, Zambia) basins, with the type locality being in the northern basin (Luhanga, Democratic Republic of Congo). These two putative species exhibit high morphological similarity. An apparent lack of morphological diversity could be attributable to non-adaptive speciation, but morphometric work would be required in order to quantify phenotypic diversity. Despite this apparent conservatism in body plan, these two *M. platysoma *clades have a relatively high genetic distance, comparable to other LT *Mastacembelus *sister species (Cyt *b *ML pairwise distances of 5.7%, compared to the mean sister species distance of 5.1%), which is also within the range recorded for other fish genera [33], e.g. *Lamprologus, Neolamprologus*.

Recent morphological work [25] split *M. albomaculatus *into two species, describing *M. reygeli *as a distinct species, whilst also suggesting the presence of intermediate populations, hypothesised to be a result of introgressive hybridisation. These authors record both *M. albomaculatus *and *M. reygeli *as being confined to the central and northern parts of the lake, with their hypothesised 'intermediate' occurring throughout the lake. The seven *M. albomaculatus *(*sensu lato*) specimens from southern LT included in our analyses would therefore have to be representative of the putative intermediate group sensu Vreven and Snoeks [25]. However, using both mtDNA and ncDNA, we found no genetic differences between the southern 'intermediate' group, and the *M. albomaculatus *from the northern part of the lake, and therefore no evidence of support for the hybridisation hypothesis of Vreven and Snoeks [25]. More extensive molecular sampling would however be useful to address the issue of hybridisation in LT mastacemblids, that has also been proposed in non-LT mastacembelids [39] but not tested. Notably, introgressive hybridisation is increasingly well documented in LT cichlids [34,40-42].

### Colonisation history of the LT mastacembelid flock

**Figure 2:**
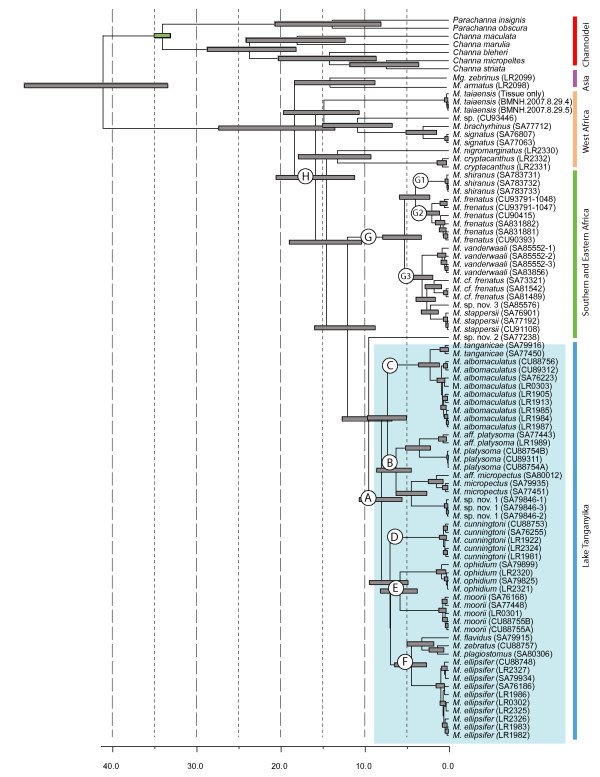
**Chronogram inferred from Bayesian dating analysis (BEAST) of the concatenated (mtDNA and ncDNA) data**. Grey node bars represent 95% confidence intervals (HPD: highest posterior distributions), the green bar represents the calibrated node. Key nodes are labelled A-H, with the blue box highlighting the LT species. Time is in millions of years (Myr).

Irrespective of dating method we find that the mastacembelid eels colonised LT ~ 7-8 Myr (BEAST analysis, 7.9 Myr, 95% HPD [highest posterior distribution]: 5.5-10.6 Myr; r8s analysis, 7.3 Myr, 95% HPD: 4.61-12.9 Myr). As the results using the two methods of dating are largely congruent, we present results from the BEAST analysis (Fig. 2). If the median dates of our estimate are correct, then *Mastacembelus *colonised and diversified in the LT basin after its formation (9-12 Myr) [1], but prior to full lacustrine conditions (5-6 Myr) [43], although the lower dating estimate occurs post deep-water conditions, corroborating the hypothesis that LT is a diversification hotspot. Our results imply that the LT *Mastacembelus *radiation pre-dates both *Synodontis *catfish 4.0-7.3 Myr [7,15] and *Platythelphusa *crabs ~2.5-3.3 Myr [6]. Dating cichlid radiations is problematic and has led to two divergent timescales, based on either a vicariance calibration linked to the breakup of Gondwana, or a calibration based on the cichlid fossil record [18], placing the cichlid diversification at 28 Myr and 12 Myr respectively. These timescales either favour a scenario in which the majority of cichlid tribes were diversifying prior to the formation of LT, or that the tribes diversified in lacustrine conditions respectively [12,41]. Although the *Lavigeria *gastropod lineage predates the formation of the LT basin [9], this genus appears to have radiated in a similar time window as LT *Mastacembelus *i.e. after the formation of the LT basin, but before full lacustrine conditions.

Diversification of the main *Mastacembelus *lineages occurred contemporaneously at ~6.2-7.2 Myr, soon after their initial seeding of the lake. This initial diversification upon colonising LT is also apparent in *Tropheus *cichlids [44], and represents a short lag time, or phylogenetic 'tail' [45]. Following the initial post-colonisation divergence, there are further contemporaneous speciation events 3-4 Myr. The split of *M. platysoma *and *M. aff. platysoma *occurs around 3.5 Myr, coinciding with the estimated formation of the southern basin of LT ~2-4 Myr [1], and a period of lower lake-levels, caused by an episode of aridification [46]. The clades (*M. micropectus, M*. sp. nov. 1) and (*M. ellipsifer (M. flavidus (M. zebratus, M. plagiostomus*))) also arose during this time of lake-level change, as do internal lineages within LT *Synodontis *[7,15] and *Platythelphusa *[6] radiations. The coinciding of speciation events in unrelated taxa with an extrinsic event, indicates that this period of lower lake-level is likely to have been a key factor responsible for promoting speciation conditions; for example, repeated segmentation and recombination of habitats along the rocky shorelines, caused by these fluctuations in water level, is likely to have resulted in the allopatric speciation of these LT radiations.

### Non-endemics

As with *Synodontis *catfish that form a LT species flock of 10 endemic plus one non-endemic species, the LT *Mastacembelus *flock is also purported to consist of endemic, plus one non-endemic species [47]. However, no specimens of the non-endemic *M. frenatus *collected from LT were available for inclusion in our analysis. The type locality of this species is the north of LT [48] and it has been recorded to occur in the catchment basin of LT, Lake Victoria and throughout the Upper Zambezi and Okavango River basins [49]. However, more recent work suggests that *M. frenatus *is not part of the LT ichthyofauna, and may not occur in the lake itself [50]. *M. frenatus *specimens from both the Malagarasi and Idete rivers in Tanzania differ markedly, and are not included within the LT flock. It therefore seems unlikely that *M. frenatus *evolved within LT and subsequently emigrated, as has been demonstrated to be the case with the non-endemic *S. victoriae*, a member of the *Synodontis *flock [7,15]. It could be reasonable to assume instead that *M. frenatus *(*sensu stricto*) as described from LT [48] may either have independently colonised the lake, or, if present in the lake at all, *M. frenatus *could represent further cryptic diversity, with the LT species being distinct from that in the surrounding rivers. Further morphological and molecular work is required in order to ascertain the diversity and taxonomy of this species complex, and its correct positioning within the group.

### African Biogeography

Analogous to Vreven [20], we found no evidence to support the genera proposed by Travers [21,23], with neither *Aeithomastacembelus *nor *Caecomastacembelus *forming monophyletic groupings (illustrated by symbols on Fig. 1). As such these names should be placed in synonymy with *Mastacembelus*. However, there is evidence for biogeographic clades. Mastacembelids have a similar distribution to other ichthyological groups, such as *Synondontis *catfish and cichlid fishes [19]. The Southern and Eastern African *Mastacembelus *species form a monophyletic group (node G on Fig. 1 and 2), consisting of three distinct biogeographic regions; i) Lake Malawi (*M. shiranus*), ii) East Africa (*M. frenatus *[Tanzania]); iii) Southern Africa (*M. vanderwaali *and *M*. sp. nov. 3 [Namibia], *M. cf. frenatus *[DR Congo and Namibia], *M. stappersii *[Zambia]). *M. signatus*, however, despite occurring in the Chambeshi River and Lake Bangwelu (both in Zambia), is resolved outside of the Southern and Eastern African clade, nesting with West African species (e.g. Cameroon, Sierra Leone). Unlike the species in the Southern-Eastern clade, *M. signatus *is not endemic to Southern/Eastern Africa, but has a distribution that extends from the Congo basin, which would therefore explain its West African affinities. It appears that *Mastacembelus *display a similar biogeographic pattern to *Synodontis*, which also form a Southern African clade [15]. Further comparative work would highlight patterns of speciation that are common to these two species-rich genera.

The ancestor to the Southern-Eastern clade appeared at ~11.9 Myr (8.7-15.9 Myr), but did not diversify until ~5 Myr (3.2-7.8 Myr) after a relatively long lag-time, which is younger than the LT flock. This could be due to incomplete taxonomic sampling, although this pattern is again evident in *Synodontis *[15], which also demonstrate a long lag-time leading to the rapid speciation within Southern Africa. This may suggest a common vicariance cause, such as changes in drainage basin structure and patterns of flow, but could be further addressed with more extensive sampling from this region. The Lake Malawi endemic, *M. shiranus*, colonised at ~3.9 Myr, post deep water conditions (4.5 Myr) [30,51]. In our analysis, West African species are recovered as basal and are paraphyletic. The non-monophyly of West African taxa may however be a consequence of our limited taxonomic sampling in this region, particularly of the lower Congo River, which has a high level of sympatric diversity, again highlighting a need for further work to more completely address the issue of regional biogeographic clades within the African *Mastacembelus*.

### Africa-Asia biogeography

The African-Asia distribution demonstrated by mastacembelid eels is a biogeographic pattern that is only observed in a few freshwater fish families, e.g. Anabantidae [52], Bagridae and Clariidae catfish [19], with the vicariance of Gondwana suggested as one possible explanation for this type of current-day distribution. However, use of this vicariance date (121-165 Myr) [53] often generates much older dates than either palaeontological or molecular data supports [e.g. [18]], and alternative hypotheses have been suggested. The African mastacembelids are monophyletic with respect to the two Asian species included, with the most recent common ancestor of the African mastacembelids dating back to ~19 Myr (13.5-27.3 Myr; Fig. 2 node H), long after the Gondwana continental break up. The median of this range estimate coincides with the closure of the Tethys Sea c. 18-20 Myr in the Early Miocene, which has been suggested as an alternative hypothesis explaining the distributions of other, albeit terrestrial, taxa [e.g. proboscideans, [54]]. The current distribution of mastacembelid eels encompasses the Middle East [19], and could be indicative of support for this hypothesis, although no samples from this region were available for inclusion in our analyses, highlighting a need for further testing in the future.

## Conclusions

The endemic LT *Mastacembelus *eel radiation is an important assemblage for studying comparative lacustrine systems, as it is divergent in life history to those already studied within the Great Lakes. The use of molecular phylogenetic techniques has revealed as yet undescribed diversity, with our data providing evidence for two potentially new LT species (*M. aff. platysoma *and *M*. sp. nov. 1). The LT *Mastacembelus *demonstrates both similarities and differences in patterns of speciation when compared to other LT radiations. For example, the origination of LT *Mastacembelus *via a single colonisation event is also demonstrated by *Platythelphusa *crabs [6] and *Cyprichromis *cichlids [12]. Using fossil calibrations from a related family, our results indicate *Mastacembelus *colonised the lake ~7.9 Myr, and is therefore an older radiation than *Synodontis *catfish, *Platythelphusa *crabs, and many cichlid tribes (e.g. Cyprichromini, Tropheini, Ectodini) if fossil dates are assumed [12]. This puts the origin of LT *Mastacembelus *within the age of the LT basin, but prior to the onset of full lacustrine conditions. Their radiation within lacustrine conditions does however further demonstrate LT as a hotspot of diversification, as opposed to an 'ancient evolutionary reservoir.' As demonstrated by other ichthyological faunas with lacustrine and fluviatile distributions (e.g. *Synodontis *catfish and cichlid fishes), our data also highlights evidence of distinct biogeographic clades. At a deeper phylogenetic level, we find evidence for an Africa-Asia split of mastacembelid eels (~19 Myr) occurring long after the divergence of the associated continents (121-165 Myr). This divergence coincides with the closure of the Tethys Sea and we therefore suggest a dispersal scenario for this group, which should be validated in the future with increased taxon sampling.

## Methods

### Taxonomic sampling

To maximise species coverage and to test species validity, samples were collected from 16 LT localities, encompassing both the southern and northern basins, resulting in 49 samples from 11 out of the 13 currently described endemic species. No DNA samples were available for *M. polli *and the newly described *M. reygeli *[25]. In order to test monophyly of the LT species flock and biogeographic scenarios a further 31 samples, from 14 non-LT African species were also included in the analyses, representing 48% of the currently described African species [19]. Two Asian mastacembelid species were included as outgroup taxa. Specimens were collected from rivers and lakes using a variety of methods, including fyke nets, scuba-diving, electro-fishing, and rotenone [55]. The latter method is particularly effective for collecting mastacembelid eels from crevices and rocky habitats (pers. obs.). Voucher numbers, collection localities, and GenBank accession numbers are listed in Additional file 1, Table S1.

### DNA extraction, PCR and sequencing

DNA was extracted from fin clips or white muscle tissue using DNeasy Blood and Tissue kit (Qiagen, UK). The use of more than one independent marker is important in order to resolve different levels of the phylogeny and provide a more complete evolutionary picture of species relationships [56,57]. Here, we sequence three molecular markers, including both mitochondrial and nuclear data: the mitochondrial genes Cyt *b *and CO1, and two introns of the ribosomal nuclear marker S7.

Cyt *b *has proved useful for elucidating both relatively deep-level and shallow time relationships in other teleosts [58], including percids [59] anabantoids [52], as well as other Lake Tanganyika species flocks [7]. It was shown to be a more informative marker for evaluating relationships of Tanganyikan *Synodontis *catfish [15] as opposed to ND2 and control region [16], although Cyt *b *can also be problematic in its use as a phylogenetic marker, e.g. in terms of saturation in the third codon position [60]. Nuclear genes are generally more slowly evolving, although the ribosomal gene S7 has been used in studies of other LT fishes, e.g. *Synodontis *catfish [15] and cichlids [34]. It has also been demonstrated to be useful at the sub-familial level, such as for the Mormyridae electric fishes [61]. The bar-coding gene CO1 [62-64], was also sequenced in order to ascertain whether *Mastacembelus *species could be delineated by this ~650 base pair mitochondrial fragment.

Published primers were used to amplify introns 1 and 2 of S7 (1224 bp aligned) [65], and the barcoding region of the CO1 gene (688 bp) [66] using annealing temperatures of 55 and 52°C respectively. Cyt b (1206 bp) was amplified using the primers MNCN-Glu F [67] and MNCN-Fish Pro R (5'-AGT TTA ATT TAG AAT YTT RGC TTT GG-3'; R Zardoya and L Rüber) using an annealing temperature of 48°C. PCR products were cleaned, and sequenced with an ABI 3730 sequencer (Applied Biosystems, UK). All sequences are deposited in GenBank, and accession numbers are given in Additional file 1, Table S1.

### Phylogenetic Analyses

The S7 sequence data were aligned in Clustal W [68] using default parameters, and indel regions were finished by eye in Se-Al [69]. Cyt *b *and CO1 sequences were aligned by eye using Se-Al.

Analyses were performed on individual gene datasets and a concatenated Cyt *b*, S7 and CO1 matrix in a total evidence approach [70]. MODELTEST v 3.7 [32] was used to ascertain the best model of molecular evolution for each dataset, selected under Akaike Information Criterion [71,72]. Variation in base composition between the taxa was assessed using the χ^2 ^test of homogeneity, implemented in PAUP* [73].

Different methods of phylogenetic reconstruction were performed in order to assess congruence between phylogenies produced by alternative methods, for both the concatenated and individual gene datasets. Maximum Likelihood (ML) analyses were performed using GARLI (Genetic Algorithm for Rapid Likelihood Inference) v0.96 [74], with model substitution rates from Modeltest applied. Bayesian Inference (BI) analyses were implemented in Mr Bayes [75], with partitions defined according to codons and genes, run for 2,000,000 generations and sampling every 100 generations, with an initial burn-in set to 5000 (chain temperature 0.2, four chains). Convergence of Metropolis-coupled Markov Chain Monte Carlo (MCMC) runs was assessed graphically using TRACER [76], and any remaining burn-in chains [77] were discarded prior to tree construction, resulting in 7500 post burn-in trees. Nodal support was ascertained with bootstrapping (BS) [78] for ML trees (1000 replicates), and Bayesian Posterior Probabilities (BPP) for the BI trees. The Approximately Unbiased (AU) [79] test as implemented in CONSEL [80] was used to test alternative topologies generated by different methods of phylogenetic inference.

### Estimation of Divergence Times

The Synbranchiformes have no fossil record that can be used in molecular dating analyses. Here, we use sequence and fossil data from a hypothesised sister group to the Synbranchiformes to provide a calibration for our study. Following Chen et al. [81], the Synbranchiformes are closely related to the Channoidei (Snakeheads) and Anabantoidae (labyrinth fishes) (Order Perciformes). The fossil record of Channoidei in Africa (genus *Parachanna*) dates back to the late Eocene [82], approximately 35-33 Myr, and this date was used to constrain the *Parachanna *node. As the fossil *Parachanna *could not be reliably assigned to any of the extant species of *Parachanna*, the calibration was placed on the stem group, rather than the crown group [83,84]. Use of a single calibration is regarded as a limitation in molecular dating [85,86], particularly when calibrations involve outgroup taxa that are some distance from the nodes of interest [87,88]. However, for studies focused at lower taxonomic levels (i.e. genus or family) where fossil data is highly limited, multiple calibrations are unrealistic. While some authors [18] have utilised vicariance dates to obtain dates of lineage divergences, such calibrations (along with lake ages) within this study would not provide an independent means of testing age of colonisation nor biogeographic scenarios. Cyt *b *sequence data from *Channa *and *Parachanna *species (Channoidei: Teleostei) were downloaded from Genbank (Additional file 1, Table S1).

The Likelihood Ratio Test [89], implemented in PAUP* with and without the molecular clock enforced, demonstrates heterogeneity in the rates of evolution across the Mastacembelidae (-ln likelihood of 21202.3 and 21077.2 respectively, ratio = 250.2, d.f. = 86, p < 0.05), and the use of non-clock-like settings (relaxed molecular clock in BEAST [90], penalised likelihood (PL) [91] method in r8s [92]) was appropriate for use in the dating analyses to convert relative molecular divergence to absolute ages. Analyses in BEAST used an uncorrelated log-normal relaxed molecular clock [93], Yule speciation prior, GTR+I+Γ model, and were run for 100,000,000 generations, with every 10,000 parameters logged. To ascertain the effect of using different priors, the analyses were repeated with the fossil constraint set to both uniform and normal distribution, and with and without partitioning the third codon. To check for the amount of burn-in, run convergence, and that the effective sample size exceed 200 for each statistic, each run was assessed graphically in Tracer v. 1.4.1 [76]. Analyses run using the uniform distribution prior on the calibrated node exhibited better stabilisation and convergence than those run with a normal distribution prior, so we present the former results. In order to compare the effects of different methods of estimating divergence times, the analysis was also run using r8s, using PL method [91,94]. The optimal smoothing parameter for the PL analysis was calculated in r8s by cross-validation and assessing the resulting χ^2 ^error. 95% confidence intervals in r8s were obtained by generating 100 bootstrap replicates and converting the topologically constrained phylograms in to penalised likelihood trees [92,95].

## Competing interests

The authors declare that they have no competing interests.

## Authors' contributions

JJD, LR and KJB designed the study. All authors collected samples, with RB collecting a substantial number of specimens used in the analyses. KJB generated molecular data and undertook the analyses. All authors read and approved the manuscript.

## Supplementary Material

Additional file 1 **Table S1. Species, collection data and GenBank accession numbers for samples used in phylogenetic analyses**. Click here for file

Additional file 2 **Phylogenetic relationships of African mastacembelid eels inferred from the Cytochrome *b *(Cyt *b*) dataset, generated using Bayesian inference**. Bayesian posterior probability values (BPP) are shown above the branch where support is >0.5. Click here for file

Additional file 3 **Phylogenetic relationships of African mastacembelid eels inferred from the Cytochrome *c *oxidase subunit 1 (CO1) dataset, generated using Bayesian inference**. Bayesian posterior probability values (BPP) are shown above the branch where support is >0.5. Click here for file

Additional file 4 **Phylogenetic relationships of African mastacembelid eels inferred from two introns of ribosomal S7, generated using Bayesian inference**. Bayesian posterior probability values (BPP) are shown above the branch where support is >0.5. Click here for file
