# Supplementary material for: Mastacembelid eels support Lake Tanganyika as an evolutionary hotspot of diversification
Source: BMC Evol Biol. 2010 Jun 19;10:188. doi: 10.1186/1471-2148-10-188 (PMC2903574; doi:10.1186/1471-2148-10-188)
Supplement: Additional file 1 — Table S1. Species, collection data and GenBank accession numbers for samples used in phylogenetic analyses. [file 1471-2148-10-188-S1.PDF]

Species	Voucher Numbers	Country	Locality	GenBank Accession numbers		
				CYTB	S7	CO1
Lake Tanganyika						
M. albomaculatus	CU 88756	TZ	Kigoma	FN812953	FN813114	FN813034
M. albomaculatus	CU 89312	TZ	Kigoma	FN812992	FN813122	FN813042
M. albomaculatus	LR 1984	ZM	Kanfonki	FN812954	FN813115	FN813039
M. albomaculatus	LR 1985	ZM	Kanfonki	FN812955	FN813116	FN813040
M. albomaculatus	LR 1987	ZM	Kanfonki	FN812960	FN813117	FN813041
M. albomaculatus	LR 0303	ZM	Mpulungu	FN812956	FN813119	FN813036
M. albomaculatus	LR 1913	ZM	Mpulungu	FN812959	FN813121	FN813037
M. albomaculatus	LR 1905	ZM	Mpulungu	FN812957	FN813120	FN813038
M. albomaculatus	SA 76223	ZM	Katoto	FN812958	FN813118	FN813035
M. cunningtoni	CU 88753	TZ	Kigoma	FN812963	FN813126	FN813044
M. cunningtoni	LR 1922	ZM	Katoto	FN812962	FN813123	FN813046
M. cunningtoni	LR 1981	ZM	Chisanza	FN812961	FN813125	FN813043
M. cunningtoni	LR 2324	ZM	Nsumbu	FN812964	FN813124	FN813045
M. cunningtoni	SA 76255	ZM	Nsumba Island	FN812965	FN813127	-
M. ellipsifer	CU 88748	TZ	Kigoma	FN813000	FN813136	FN813056
M. ellipsifer	LR 0302	ZM	Nkumbula Island	-	FN813131	FN813054
M. ellipsifer	LR 1982	ZM	Misepa	FN812966	FN813128	FN813047
M. ellipsifer	LR 1983	ZM	Misepa	FN812967	FN813133	FN813048
M. ellipsifer	LR 1986	ZM	Kanfonki	FN812968	FN813132	FN813049
M. ellipsifer	LR 2325	ZM	Nsumbu	FN812969	FN813129	FN813050
M. ellipsifer	LR 2326	ZM	Nsumbu	FN812970	FN813134	FN813051
M. ellipsifer	LR 2327	ZM	Nsumbu	FN812971	FN813130	FN813052
M. ellipsifer	SA 76186	ZM	Musende Rocks	FN812994	FN813135	FN813055
M. ellipsifer	SA 79934	ZM	Mbita Island	FN812972	-	FN813053
M. flavidus	SA 79915	ZM	Onzye Point	FN812973	-	FN813057
M. micropectus	SA 77451	ZM	Mbita Island	FN812996	-	FN813059
M. micropectus	SA 79935	ZM	Mbita Island	FN812974	FN813137	FN813058
M. cf. micropectus	SA 80012	ZM	Chimba	FN812995	FN813138	FN813060
M. moorii	CU 88755A	TZ	Kigoma	FN812976	FN813139	FN813062
M. moorii	CU 88755B	TZ	Kigoma	FN812978	FN813140	FN813063
M. moorii	LR 0301	ZM	Mpulungu	FN812975	FN813142	FN813061
M. moorii	SA 76168	ZM	Mbita Island	FN812993	FN813141	FN813065
M. moorii	SA 77448	ZM	Mbita Island	FN812977	-	FN813064
M. ophidium	LR 2320	ZM	Ndole Bay	FN812979	FN813143	FN813066
M. ophidium	LR 2321	ZM	Ndole Bay	FN812980	-	FN813067
M. ophidium	SA 79825	ZM	Cape Kashese	FN812981	FN813144	FN813068
M. ophidium	SA 79899	ZM	Musende Bay	FN812982	FN813145	FN813069
M. plagiostomus	SA 80306	ZM	Kombe	FN812984	FN813149	FN813070
M. platysoma	CU 88754A	TZ	Kigoma	FN812987	FN813147	FN813074
M. platysoma	CU 88754B	TZ	Kigoma	FN812988	FN813148	FN813075
M. platysoma	CU 89311	TZ	Kigoma	FN812991	FN813152	FN813076
M. aff. platysoma	LR 1989	ZM	Kanfonki	FN812985	FN813146	FN813072
M. aff. platysoma	SA 77443	ZM	Mbita Island	FN812986	-	FN813073
M. sp. nov. 1	SA 79846-1	ZM	Cape Kashese	FN812997	FN813153	FN813079
M. sp. nov. 1	SA 79846-2	ZM	Cape Kashese	FN812998	FN813154	FN813080
M. sp. nov. 1	SA 79846-3	ZM	Cape Kashese	FN812999	FN813155	FN813081
M. tanganicae	SA 77450	ZM	Mbita Island	FN812989	FN813150	FN813077
M. tanganicae	SA 79916	ZM	Onzye Point	-	-	FN813078
M. zebratus	CU 88757	TZ	Kigoma	FN812990	FN813151	FN813071
Rest of Africa						
M. brachyrhinus	SA 77712	CAR	Baidou River	FN813017	FN813178	FN813110
M. cryptacanthus	LR 2331	BN	Oueme Delta	FN813015	FN813182	FN813108
M. cryptacanthus	LR 2332	BN	Iguidi/Langbe	FN813016	FN813183	FN813109
M. frenatus	CU 93791-1047	TZ	Idete River	FN813001	FN813161	FN813085
M. frenatus	CU 93791-1048	TZ	Idete River	FN813002	FN813162	FN813086
M. frenatus	CU 90393	TZ	Malagarasi River	FN813009	FN813159	FN813087
M. frenatus	CU 90415	TZ	Malagarasi River	FN813010	FN813160	FN813088
M. frenatus	SA 83188-1	TZ	Malagarasi River	FN813003	FN813164	FN813083
M. frenatus	SA 83188-2	TZ	Malagarasi River	FN813004	FN813163	FN813084
M. cf. frenatus	SA 81489	DRC	Luapula River	FN813023	FN813165	FN813089
M. cf. frenatus	SA 81542	DRC	Luapula River	FN813022	FN813166	FN813090
M. cf. frenatus	SA 73321	ZM	Maheba River	FN813005	-	-
M. nigromarginatus	LR 2330	BF	Bougouriba, Drebougou	FN813027	FN813172	FN813099
M. shiranus	SA 78373-1	MW	Rukuru, Lake Malawi	FN813006	FN813158	FN813091
M. shiranus	SA 78373-2	MW	Rukuru, Lake Malawi	FN813007	FN813156	FN813092
M. shiranus	SA 78373-3	MW	Rukuru, Lake Malawi	FN813008	FN813157	FN813093
M. signatus	SA 76807	ZM	Lake Bangweulu	FN813025	FN813173	FN813100
M. signatus	SA 77063	ZM	Chambeshi River	FN813020	-	FN813101
M. stappersii	SA 76901	ZM	Luongo River	FN813026	FN813175	FN813102
M. stappersii	SA 77192	ZM	Kalungwishi River	FN813021	FN813176	FN813103
M. stappersii	CU 91108	ZM	Lufubu River	FN813019	FN813174	FN813104

Species	Voucher Numbers	Country	Locality	GenBank Accession numbers		
				CYTB	S7	CO1
M. taiaensis	Tissue only (JJD5519)	SL	Bumbuna, Rokel River	FN813012	FN813179	FN813105
M. taiaensis	BMNH 2007.8.29.4	SL	Bumbuna, Rokel River	FN813013	FN813180	FN813106
M. taiaensis	BMNH 2007.8.29.5	SL	Bumbuna, Rokel River	FN813014	FN813181	FN813107
M. vanderwaali	SA 85552-1	NB	Katima, Zambezi River	FN813028	FN813167	FN813094
M. vanderwaali	SA 85552-2	NB	Katima, Zambezi River	FN813029	FN813168	FN813095
M. vanderwaali	SA 85552-3	NB	Katima, Zambezi River	FN813030	FN813169	FN813096
M. vanderwaali	SA 83856	NB	Katima, Zambezi River	FN813031	FN813170	FN813097
M. sp.	CU 93446	CR	Djerem River	FN813024	FN813184	FN813111
M. sp. nov. 2	SA 77238	ZM	Kalungwishi River	FN813018	FN813177	FN813082
M. sp. nov. 3	SA 85576	NB	Popa Falls	FN813011	FN813171	FN813098
Asian outgroups						
M. armatus	LR 2098	MY	-	FN813032	FN813185	FN813112
Macrognathus zebrinus	LR 2099	MY	-	FN813033	FN813186	FN813113
Outgroups for dating						
Channa bleheri	N/A	N/A	N/A	AY763770	-	-
Channa maculata	N/A	N/A	N/A	AF479271	-	-
Channa marulia	N/A	N/A	N/A	AY763771	-	-
Channa micropeltes	N/A	N/A	N/A	AF012785	-	-
Channa striata	N/A	N/A	N/A	AF012789	-	-
Parachanna insignis	N/A	N/A	N/A	Unpublished	-	-
Parachanna obscura	N/A	N/A	N/A	AY763772	-	-

Abbreviations: *M.*, *Mastacembelus*; BMNH, The Natural History Museum, London; SA, South African Institute of Aquatic Biodiversity (SAIAB); CU, Cornell University Museum of Zoology; LR, collected by Lukas Rüber (held at Natural History Museum, London).

Country codes: BF, Burkina Faso; BN, Benin; CAR, Central African Republic; CR, Cameroon; DRC, Democratic Republic of Congo; MW, Malawi; MY, Myanmar; NB, Namibia; SL, Sierra Leone; TZ, Tanzania; ZM, Zambia.

Lake Tanganyika locality name variations: Sumba Island, Sumbu Island; Mbita Island, Nkumbula Island; Onzye Point, Wonzye Point; Cape Kashese, Cape Kachese.
