# Supplementary figures and images for: Mastacembelid eels support Lake Tanganyika as an evolutionary hotspot of diversification
Source: BMC Evol Biol. 2010 Jun 19;10:188. doi: 10.1186/1471-2148-10-188 (PMC2903574; doi:10.1186/1471-2148-10-188)

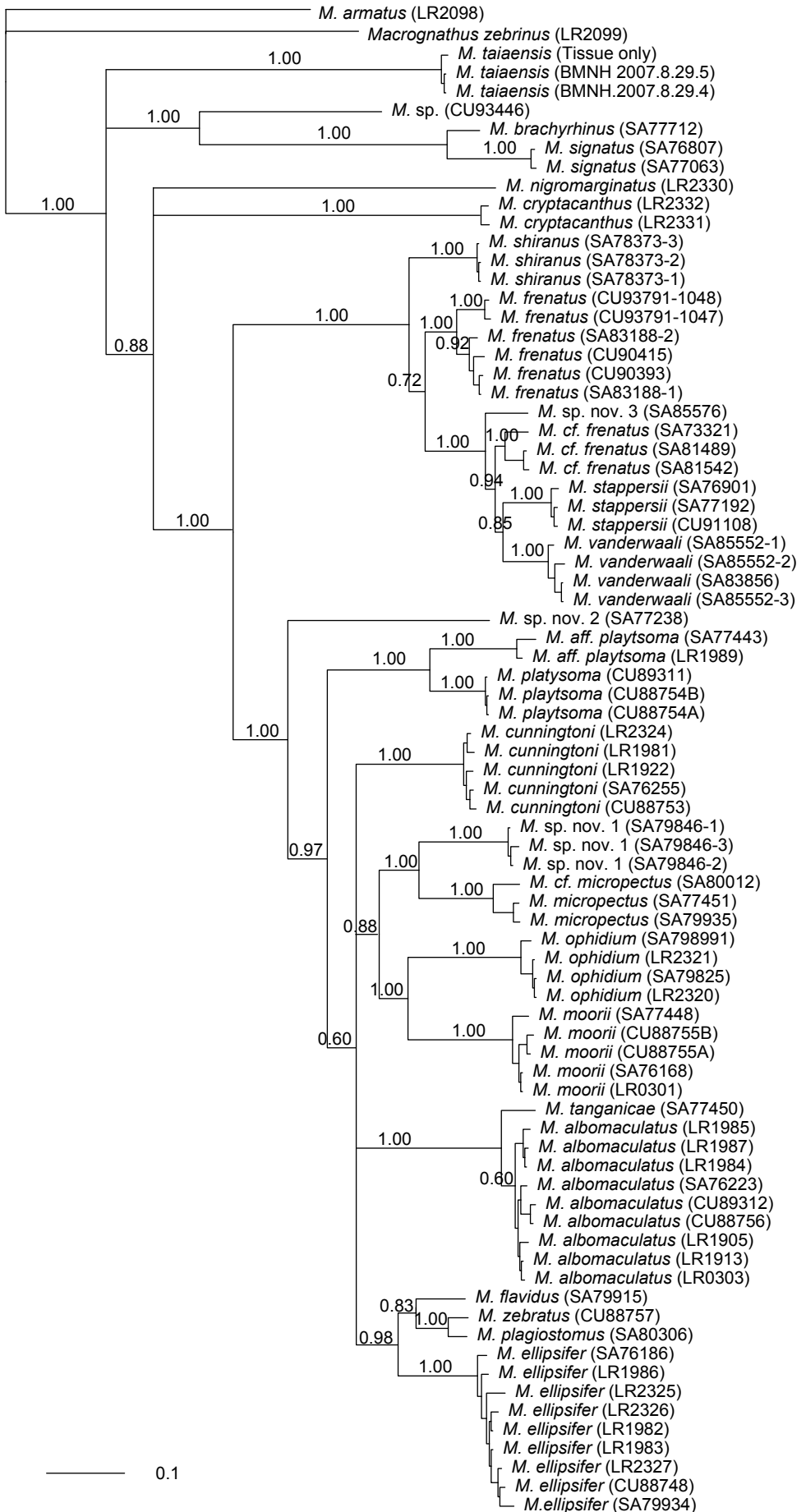

Supplement: Additional file 2 — Phylogenetic relationships of African mastacembelid eels inferred from the Cytochrome b (Cyt b) dataset, generated using Bayesian inference. Bayesian posterior probability values (BPP) are shown above the branch where support is >0.5. [file 1471-2148-10-188-S2.PDF]

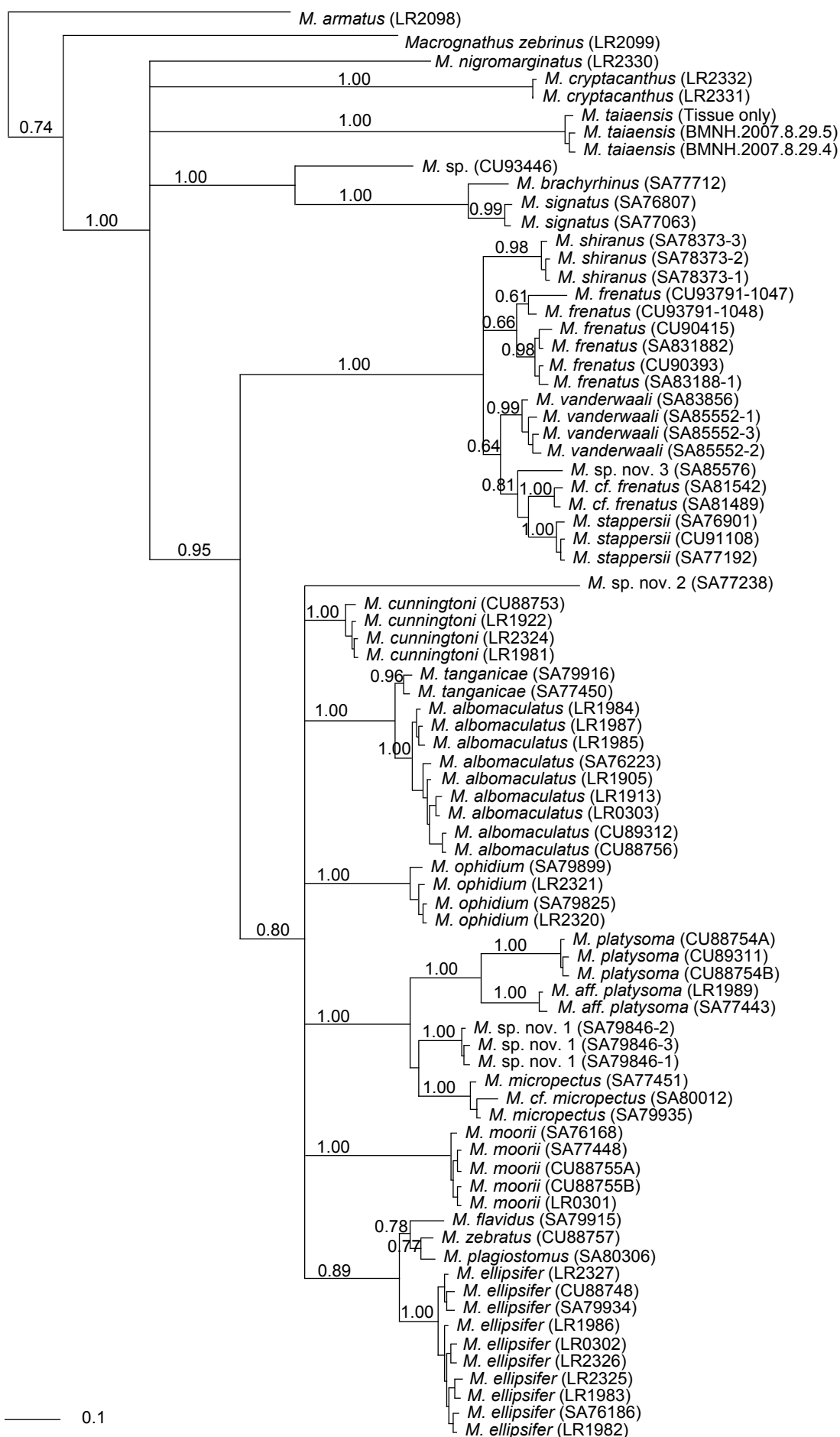

Supplement: Additional file 3 — Phylogenetic relationships of African mastacembelid eels inferred from the Cytochrome c oxidase subunit 1 (CO1) dataset, generated using Bayesian inference. Bayesian posterior probability values (BPP) are shown above the branch where support is >0.5. [file 1471-2148-10-188-S3.PDF]

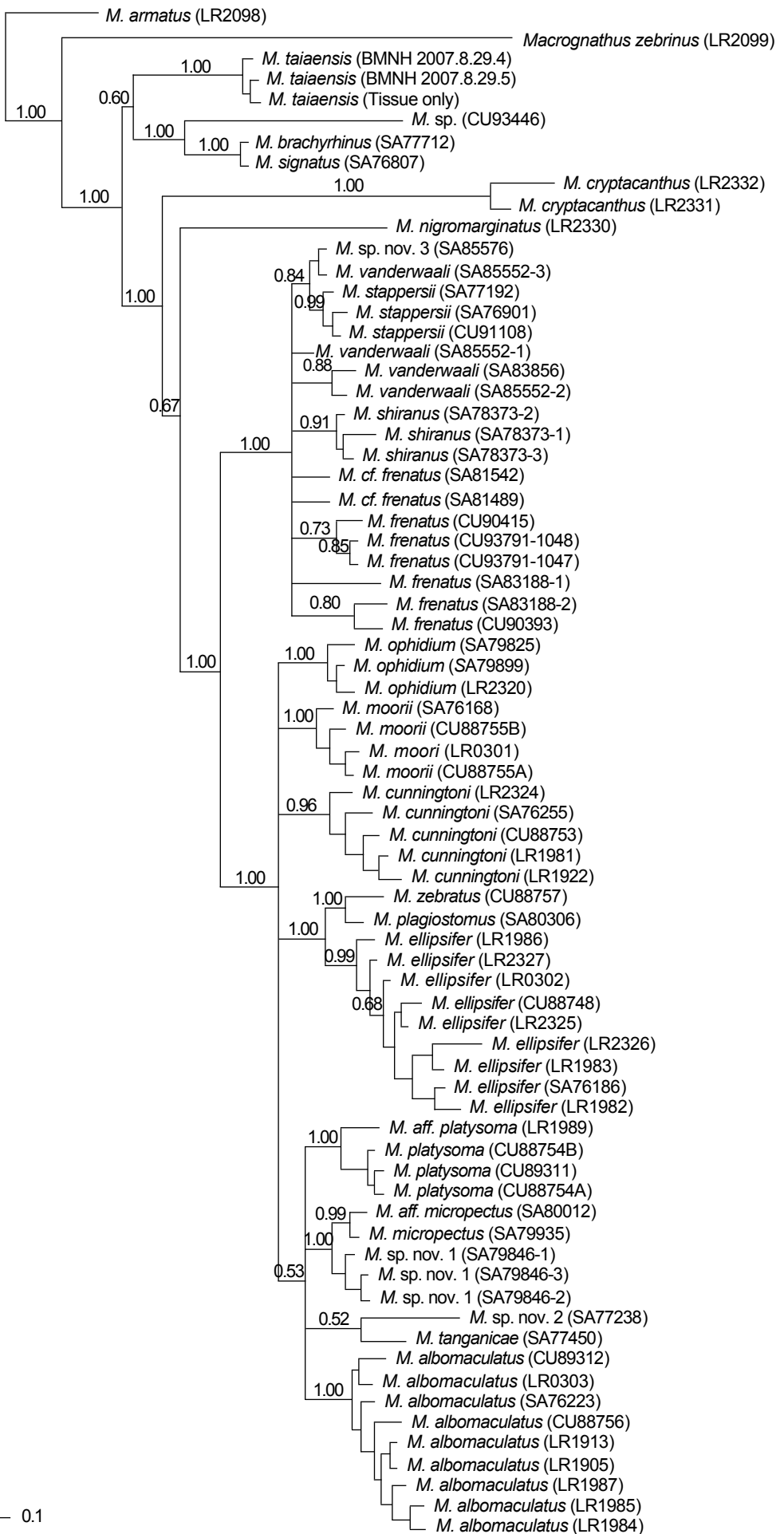

Supplement: Additional file 4 — Phylogenetic relationships of African mastacembelid eels inferred from two introns of ribosomal S7, generated using Bayesian inference. Bayesian posterior probability values (BPP) are shown above the branch where support is >0.5. [file 1471-2148-10-188-S4.PDF]
